# ASCOT ADAPT study of COVID-19 therapeutics in hospitalised patients: an international multicentre adaptive platform trial

**DOI:** 10.1186/s13063-022-06929-y

**Published:** 2022-12-14

**Authors:** Justin T. Denholm, Balasubramanian Venkatesh, Joshua Davis, Asha C. Bowen, Naomi E. Hammond, Vivekanand Jha, Grace McPhee, Zoe McQuilten, Matthew V. N. O’Sullivan, David Paterson, David Price, Megan Rees, Jason Roberts, Mark Jones, James Totterdell, Thomas Snelling, Nanette Trask, Susan Morpeth, Steven YC Tong, on behalf of the ASCOT ADAPT investigators

**Affiliations:** 1 Victorian Infectious Diseases Service, The Royal Melbourne Hospital, at the Peter Doherty Institute for Infection and Immunity, Melbourne, Australia; 2 Department of Infectious Diseases, The University of Melbourne at the Peter Doherty Institute for Infection and Immunity, Melbourne, Victoria 3000 Australia; 3 The George Institute for Global Health, Sydney, Australia; 4 The George Institute for Global Health, New Delhi, India; 5 Faculty of Medicine, University of New South Wales, Sydney, Australia; 6 Menzies School of Health Research, Charles Darwin University, Darwin, Australia; 7 Department of Infectious Diseases, John Hunter Hospital, Newcastle, NSW Australia; 8 Department of Infectious Diseases, Perth Children’s Hospital, Perth, Australia; 9 Wesfarmers Centre for Vaccines and Infectious Diseases, Telethon Kids Institute, University of Western Australia, Perth, Australia; 10 Australian and New Zealand Intensive Care Research Centre (ANZIC-RC), Monash University, Melbourne, Australia; 11 Transfusion Research Unit, Monash University, Melbourne, Australia; 12 Department of Infectious Diseases Westmead Hospital, Westmead, Australia; 13 NSW Health Pathology, Institute for Clinical Pathology and Medical Research, Westmead, Australia; 14 Sydney Institute for Infectious Diseases, University of Sydney, Sydney, Australia; 15 University of Queensland Centre for Clinical Research, Faculty of Medicine & Centre for Translational Anti-infective Pharmacodynamics, School of Pharmacy, The University of Queensland, Brisbane, Australia; 16 Department of Infectious Diseases, Royal Brisbane and Women’s Hospital, Brisbane, Queensland Australia; 17 Department of Respiratory Medicine, The Royal Melbourne Hospital and Department of Medicine, University of Melbourne, Melbourne, Victoria Australia; 18 Departments of Pharmacy and Intensive Care Medicine, Royal Brisbane and Women’s Hospital, Brisbane, Australia; 19 Division of Anaesthesiology Critical Care Emergency and Pain Medicine, Nîmes University Hospital, University of Montpellier, Nîmes, France; 20 School of Public Health, University of Sydney, Camperdown, Australia; 21 Perth, Australia; 22 Middlemore Hospital, Counties Manukau District Health Board, Auckland, New Zealand

**Keywords:** COVID-19, SARS-CoV-2, Randomised controlled trial, Antiviral medication, Anticoagulation, Antibody

## Abstract

**Background:**

SARS-CoV-2 infection is associated with a significant risk of hospitalisation, death, and prolonged impact on quality of life. Evaluation of new treatment options and optimising therapeutic management of people hospitalised with SARS-CoV-2 infection remains essential, but rapid changes in pandemic conditions and potential therapies have limited the utility of traditional approaches to randomised controlled trials.

**Methods:**

ASCOT ADAPT is an international, investigator-initiated, adaptive platform, randomised controlled trial of therapeutics for non-critically ill patients hospitalised with COVID-19. The study design is open label and pragmatic. Potential participants are hospitalised adults with PCR confirmed, symptomatic, SARS-CoV-2 infection, within 14 days of symptom onset. Domains include antiviral, antibody and anticoagulant interventions, with a composite primary outcome of 28-day mortality or progression to intensive-care level respiratory or haemodynamic support. Initial interventions include intravenous nafamostat and variable dose anticoagulation. A range of secondary endpoints, and substudies for specific domains and interventions are outlined.

**Discussion:**

This paper presents the trial protocol and management structure, including international governance, remote site monitoring and biobanking activities and provides commentary on ethical and pragmatic considerations in establishing the ASCOT ADAPT trial under pandemic conditions.

**Trial registration:**

Australian and New Zealand Clinical Trials Registry (ACTRN12620000445976) and ClinicalTrials.gov (NCT04483960).

**Supplementary Information:**

The online version contains supplementary material available at 10.1186/s13063-022-06929-y.

## Background

The development of COVID-19 vaccines with efficacy against both infection and severe disease is a welcome and remarkable development and key to pandemic control [1, 2]. Despite vaccine availability, optimising therapeutics for people hospitalised with COVID-19 remains essential as existing vaccines provide imperfect protection and because access to vaccines remains limited in many countries [3]. The emergence of SARS-Co-V variant strains associated with apparent differential vaccine efficacy is another reason why therapeutics will remain critical for the foreseeable future.

Initially, the ASCOT trial was developed as a 2 × 2 factorial trial of COVID-19 therapeutics [4]. This design was selected in the early COVID-19 pandemic period for relative simplicity and allowed the rapid establishment of a network of active trial sites across Australia and New Zealand. From the outset, however, it was intended that this be used as a forerunner to the development of ASCOT ADAptive Platform Trial (ADAPT), which is outlined in this report.

## Methods

Adaptive Platform Trials are an innovative trials methodology [5–7], which have been successfully operationalised by RECOVERY [8–10], SOLIDARITY [11], and REMAP-CAP [12, 13], arguably the clinical trials that have most advanced COVID-19 clinical therapeutics to date.

Conventional randomised controlled trials (RCT), at the time of design, make assumptions about plausible effect size, primary outcome incidence, and sample size; holding assumptions constant until trial completion. Adaptive trials incorporate pre-specified adaptation mechanisms, which allows their structure to evolve based on accruing evidence. Platform trials are adaptive trials that allow multiple questions to be evaluated simultaneously with the goal of identifying optimal treatments for a disease as efficiently as possible.

ASCOT ADAPT comprises the following critical components:

- A core master protocol with minimal, easily identified inclusion and exclusion criteria, specifying the key primary endpoint of 28-day mortality or need for intensive respiratory support
- A number of parallel treatment domains, initially including antiviral, antibody and anticoagulation interventions
- Frequent sequential analyses and pre-specified stopping rules for effectiveness, superiority, futility and inferiority in each domain
- Bayesian hierarchical modelling to allow information sharing across pre-specified patient groups
- Response adaptive randomisation to allow increased allocation to better performing treatment arms within each domain during the course of the trial. Randomisation occurs within a centralised platform database (Spinnaker), with site investigators enrolling patients sequentially at participating hospitals
- Potential to extend trial duration indefinitely provided appropriate clinical questions persist and funding support is available

**Fig. 1:**
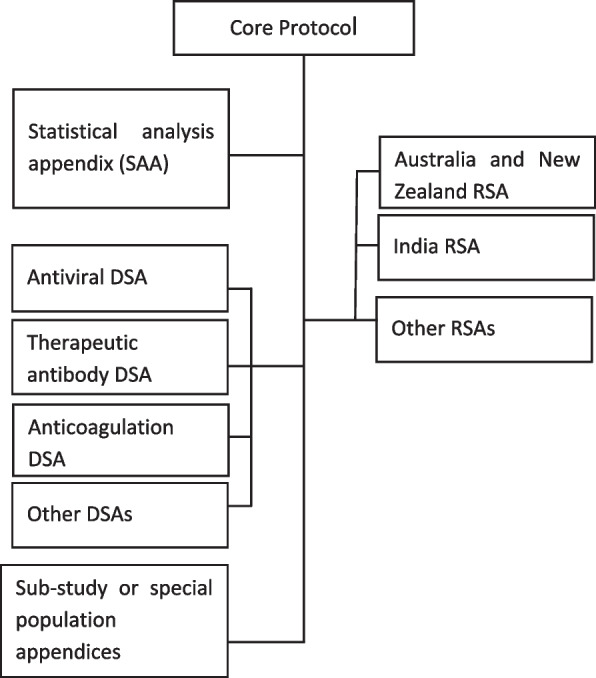
Patient flow if eligible for platform entry

The protocol documents for ASCOT ADAPT consist of an overarching core protocol, a statistical appendix, a domain specific appendix (DSA) for each domain and region-specific appendices (RSA) (Fig. 1). The protocol is designed to minimise the need for core amendments or ethical review with trial adaptations (for example, the addition of interventions within a domain, or of entire domains). Trial documents, including core and domain protocols and statistical appendices, are available at https://www.ascot-trial.edu.au/pages/resources

**Fig. 2:**
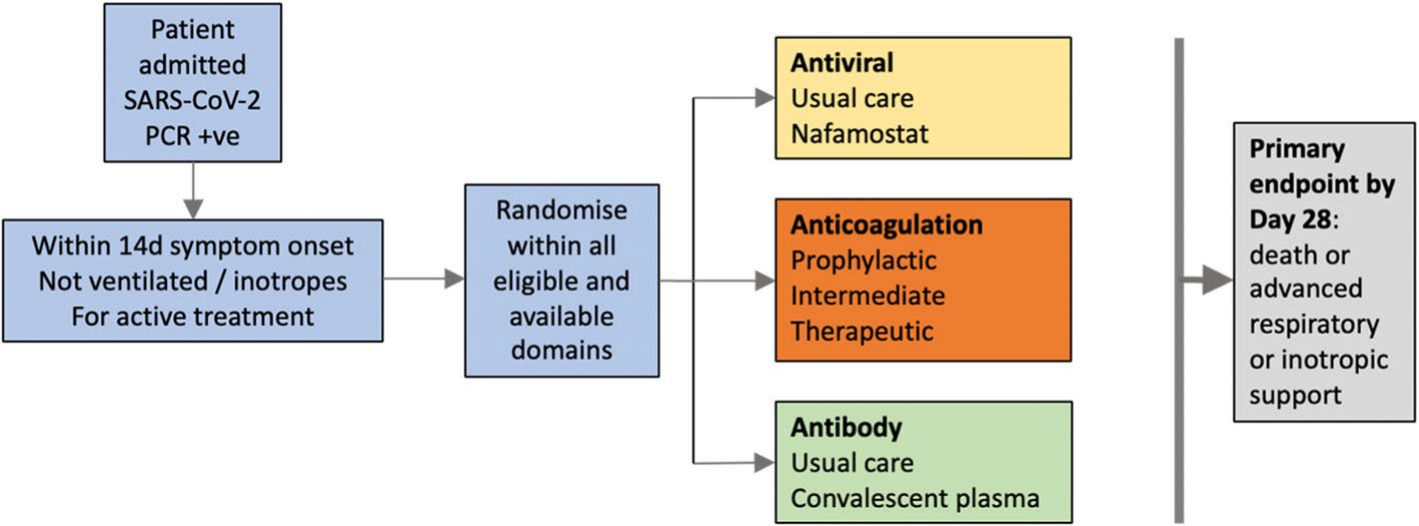
Three domains of ASCOT ADAPT

ASCOT ADAPT commenced with three domains, with the potential to add more domains in the future. In theory, an unlimited number of domains are possible but must be balanced by pragmatic restrictions on trial size and conduct. These are antiviral, therapeutic antibody and anticoagulation domains (Fig. 2). An important aspect of the pragmatic design of ASCOT ADAPT is the recognition that “standard of care” for COVID-19 will evolve during the life of the platform and that patients enrolled in ASCOT-ADAPT will be allowed to receive standard of care interventions in addition to allocated trial interventions, as long as there is no biological interaction or contraindication with an active domain. In other words, ASCOT ADAPT is testing the effect of the addition of selected interventions to the current standard of care, seeking an incremental benefit. At the time of writing, standard of care for severe COVID-19 in Australia includes low-dose thromboprophylaxis, dexamethasone, remdesivir (if on supplemental oxygen but not mechanically ventilated), and one of baricitinib, tocilizumab, or sarilumab [14].

ASCOT ADAPT discourages the use of therapies which have not clearly been shown to provide clinical benefit but does not exclude participants from enrolment if they have been administered. Initial domains and interventions are described in detail below, and new interventions can be added to them and other domains if and when they become available and are assessed as meeting criteria to enter the platform. To be added to the platform, a candidate intervention is first assessed by the ASCOT ADAPT therapeutics advisory committee, who may seek further information and can then recommend an intervention to the global trial steering committee for addition to the platform. In general, to be added to the platform, an intervention needs to have sufficient human safety data, a strong biological plausibility for activity against SARS-CoV-2 or the associated host response and have preliminary human efficacy data. In addition, ASCOT ADAPT will prioritise interventions which are available and affordable.

**Fig. 3:**
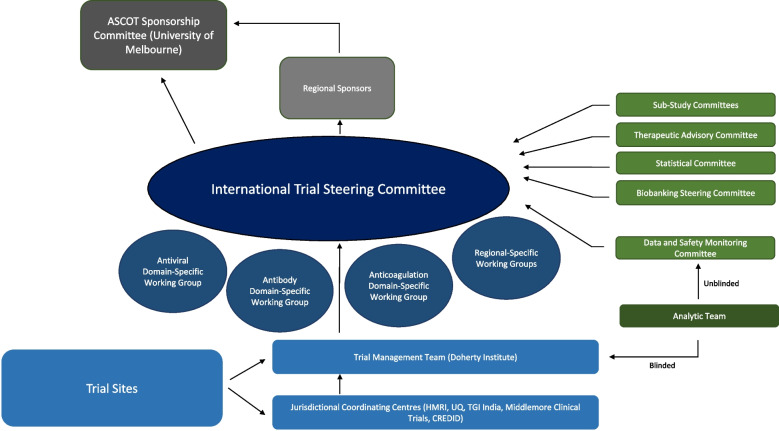
Key governance structures overseeing the trial implementation and conduct

The overall aim of the ASCOT ADAPT study is to evaluate potential therapeutics in adults hospitalised with SARS-CoV-2 infection, for reducing all-cause mortality or progression to intensive care level support within 28 days of randomisation. The key elements of the trial design are summarised in Table 1. The SPIRIT checklist is attached as Additional file 1 [15]. Figure 3 shows key governance structures overseeing the trial implementation and conduct.

**Table 1:** Key trial design elements for ASCOT ADAPT

Core primary outcome measure	Death from any cause or requirement of new intensive respiratory support (invasive or non-invasive ventilation) or vasopressor/inotropic support in the 28 days after randomisation
**Core secondary outcome measures**	1. Time to clinical recovery during the first 28 days after randomisation 2. WHO 8-point ordinal outcome scale at day 28 post randomisation 3. All-cause mortality at 28 and 90 days post randomisation 4. Days alive and free of hospital by 28 days post randomisation 5. Days alive and free of ventilation by 28 days post randomisation 6. Presence of patient reported outcome of shortness of breath at days 28 and 90 post randomisation 7. Quality of life as measured by EQ5D5L questionnaire at days 28 and 90 post randomisation
**Study domains**	1. Antiviral domain 2. Therapeutic antibody domain 3. Anticoagulation domain 4. Other domain (to be determined)
**Platform inclusion criteria**	1. Age ≥ 18 years 2. Admitted to an acute-care hospital 3. Confirmed SARS-CoV-2 by nucleic acid testing in the 14 days prior to randomisation 4. Able to be randomised within 14 days of symptom onset 5. At least one symptom or sign attributable to SARS-CoV-2 infection
**Platform exclusion criteria**	1. Currently receiving acute intensive respiratory support (invasive or non-invasive ventilation) or vasopressor/inotropic support 2. Previous participation in the trial 3. Treating team deems enrolment in the study is not in the best interest of the patient 4. Death is deemed to be imminent and inevitable within the next 24 h 5. Either the patient or their primary treating clinician are not committed to active treatment

### Study sites, participants and endpoints

At the time of writing, ASCOT ADAPT is operational in centres across Australia, New Zealand and India. Sites are nominated by local investigators and selected by a process involving assessment of local capacity. Efforts have been made to ensure available sites in areas of active COVID-19 community transmission to maximise study access, but support has also been provided for establishing sites in areas with little or no COVID-19 activity in anticipation of future need. Regular investigator meetings are held by teleconference, including updates on planned and newly introduced domains and interventions, and supplemented by weekly email updates.

In all sites, participants are hospitalised adult patients with confirmed SARS-CoV-2. This participant cohort was chosen for a variety of reasons, including their high risk for progression to intensive care support and/or death, and the limited therapeutic portfolio currently available to improve disease outcomes. The primary endpoint is death from any cause or requirement of new intensive respiratory support (invasive or non-invasive ventilation) or vasopressor/inotropic support in the 28 days after randomisation. This primary endpoint was chosen to reflect objective and highly prioritised outcomes, and to align with other international studies [16]. Additional secondary endpoints were also chosen to reflect community priorities resulting from focus group meetings with a wide range of stakeholders [17].

Recruitment opened on 12 February 2021. As of 1 October 2021, 1075 participants had been enrolled.

The ASCOT ADAPT trial is registered with the Australian and New Zealand Clinical Trials Registry (ACTRN12620000445976) and ClinicalTrials.gov (NCT04483960).

### Domains and interventions

ASCOT ADAPT began with antiviral, antibody, and anticoagulant domains.

*The antiviral domain* was planned to start by comparing interferon-β, interferon-β plus ribavirin, nafamostat and standard of care arms. The interferon arms were based on biological plausibility, an in vitro effect against SARS CoV-2 and clinical trial data among patients with COVID-19 from Hong Kong [18]. However, when the WHO Solidarity trial reported that interferon had no effect on the course of COVID-19, the decision was made by the Trial Steering Committee (TSC) in consultation with the Data Safety and Monitoring Committee (DSMC) to drop these arms and focus on nafamostat [11].

Nafamostat is a serine protease inhibitor. It inhibits TMPRSS2, the serine protease that primes the spike protein as part of SARS-COV-2 entry into human cells [19]. It is also an antifibrinolytic agent with weak anticoagulant properties used for prevention of coagulation of perfused blood during extracorporeal blood circulation for dialysis and is licensed in Japan and Korea for this indication and for pancreatitis. The antifibrinolytic properties of nafamostat mean that it has been used in the treatment of disseminated intravascular coagulation (DIC)—a further potential advantage of this drug as DIC can be a complication of COVID-19 [20]—and in the maintenance of cardiopulmonary bypass in patients with recent intracerebral haemorrhage [21].

Nafamostat has potent anti-SARS-CoV-2 activity in a human epithelial cell line CALU-3 [22]. The EC50 is 5-10 nM and estimated EC90 of 47nM (extrapolated from Hoffman et al). This potency exceeds that of almost all other compounds being tested as potential antiviral therapies against SARS-CoV-2, including remdesivir, making it a highly attractive compound to study. Very few drugs have low nanomolar EC50 values.

Nafamostat has moderate volume of distribution ~5 L and protein binding of 67%. Although specific data are lacking, these pharmacokinetic characteristics suggest nafamostat has extensive tissue penetration and concentrations should be favourable in highly vascularised tissues like the lung. Nafamostat is hydrolysed in blood and is not metabolised by the liver and so is not likely to be a cause of hepatic enzymosis nor would hepatic dysfunction theoretically preclude use. The half-life is very short (in the order of minutes), and so nafamostat must be administered by constant intravenous infusion. A continuous infusion of 0.2 mg/kg/h is estimated to result in steady-state total drug concentrations of 170–200 nM. When accounting for protein binding, this means the steady-state concentration: EC50 ratio is 11.2–13.2 and EC90 ratio is estimated to be 1.2–1.4. Nafamostat has been used in small case series of patients hospitalised with COVID-19 and anecdotally is in common use for this indication in Japan [23], but RCT evidence of efficacy is needed [24, 25].

*The antibody domain* initially studied convalescent plasma, with the capacity to incorporate the addition of hyperimmune globulin, monoclonal antibodies or other therapeutic antibodies. At the time, convalescent plasma looked like a promising treatment for COVID-19 based on observational data [26, 27]. The USA FDA had issued an emergency use authorisation resulting in widespread uptake of use of convalescent plasma therapeutically without solid RCT evidence. The RECOVERY trial subsequently reported futility in the use of convalescent plasma in the treatment of hospitalised patients with COVID-19 [28], as has the REMAP-CAP trial among critically unwell patients admitted to ICU with COVID-19 [29]. A decision was then made by the ASCOT-ADAPT TSC and DSMC to close convalescent plasma on 4 February 2021 as an intervention after 33 patients had been randomised within the antibody domain. The domain remains suspended at the time of writing.

*The anticoagulation domain* is currently studying prophylactic dose low molecular weight heparin (LMWH), which was the standard of care treatment for hospitalised patients with COVID-19, compared with intermediate dose LMWH and with therapeutic anticoagulation. Intermediate dose LMWH is in between traditional prophylactic dosing and therapeutic anticoagulation dosing of LMWH or intravenous heparin. A previous protocol was amended to remove a prophylactic dose + aspirin arm, following results from REMAP-CAP and RECOVERY demonstrating that aspirin provided no evident clinical benefit [30]. Approximately 280 patients had been enrolled and assigned to prophylactic dose + aspirin.

The propensity of SARS-CoV-2 to cause microvascular, venous, and arterial thrombosis appears to be both part of the pathogenesis of the disease and to cause complications among patients with moderate to severe COVID-19 [31]. The multi-platform consortium of REMAP-CAP, ACTIV, and ATTACC have recently reported on a comparison between standard prophylaxis and therapeutic anticoagulation for patients with moderate and severe COVID-19 [32, 33]. They found that therapeutic anticoagulation was beneficial for patients admitted to hospital with moderate COVID-19, but not for patients in ICU requiring critical care for COVID-19. They did not evaluate intermediate dose LMWH, which as pointed out in the accompanying editorial, remains an important question to resolve [34]. The question of whether therapeutic anticoagulation is beneficial for patients such as those who will be enrolled in ASCOT ADAPT would also be important to ascertain in a low-middle income country such as India or Nepal.

### Data collection and management

In keeping with a clinical trial implemented under pandemic conditions and elevated health system pressures, a pragmatic and streamlined core dataset was developed. Data collection is conducted by trained staff at each participating site and entered into an electronic case report form. Information collected includes eligibility criteria at randomisation, baseline patient demographic and medical information (to evaluate balance of randomisation), and information to categorise patients into the a priori subgroups of interest. During the first 14 days while the patient remains in hospital, information on daily clinical parameters is collected to measure secondary outcomes and ensure protocol compliance. At 28 days post-randomisation, information on vital status is obtained. At 28 and 90 days following randomisation, quality of life is measured using the EQ5D-5L score.

### Statistical considerations

As detailed in Table 1, the core primary outcome measure is death from any cause or requirement of new intensive respiratory support (invasive or non-invasive ventilation) or vasopressor/inotropic support in the 28 days after randomisation, represented as a binary random variable. The objective of ASCOT ADAPT is to evaluate therapeutics in a generalised factorial structure (conceptualised as domains) relative to both standard of care and all other treatments within a domain. We characterise treatment efficacy in terms of a continuum from benefit to futility. Specifically, we define ‘effective’ treatments as those that lower the log-odds of the primary outcome and ‘futile’ treatments as those that reduce the log-odds by no more than -log(1.1). A superior treatment is defined as one which has a lower log-odds than all other interventions in that domain.

Bayesian logistic regression models are used to evaluate the efficacy of treatment regimens (distinct combinations of treatments across domains) with respect to the primary outcome, adjusting for region, site, age, and time since trial commencement. Sequential analyses are scheduled to occur approximately every 3rd month throughout the trial. Each analysis uses data on all participants who have 28-day outcome data available to estimate the log-odds of the primary endpoint for all randomised participants irrespective of post-randomisation events (intent-to-treat principle). To account for temporal trends, participants are grouped into sequential cohorts (4-week epochs), with the effect of time modelled as a random walk with a prior that enforces smoothing of time-effects across adjacent epochs. Bayesian inferences are made from Markov chain Monte Carlo (MCMC) draws from the joint posterior distribution of model parameters.

At each analysis, we contrast the primary estimand for each treatment arm with standard of care in its domain. If at any analysis a treatment is estimated to be:

- Futile with > 95% probability, it will be dropped
- Effective with > 99% probability, the standard of care will be dropped
- Superior with > 99% probability, all other treatments in that domain will be dropped

Response adaptive randomisation is centrally implemented for dynamically allocating treatments via a secure proprietary web interface. Following each analysis, the targeted allocation probabilities (by regimen) will be updated such that they are proportional to the probability that each regimen minimises the log-odds of the outcome. Where there are more than two interventions, allocation to usual care within a domain will be fixed as the reciprocal of the number of interventions in the domain. If the domain includes only usual care and one other active intervention, then a minimum allocation probability of 1/3 will be targeted to either intervention within that domain.

Secondary analysis methods include binary, ordinal and time to event models, all of which are implemented with a Bayesian logistic model framework that is extended as required. For ordinal measures, proportional odds are assumed.

Pre-specified subgroups are defined to explore treatment effect heterogeneity and sensitivity analyses examine how model estimates vary conditional on pre-specified analysis sets (intent-to-treat, per protocol, contemporaneous reference groups), independent domain modelling, prior specification and the implications of missing data.

A complete statistical appendix is available at https://www.ascot-trial.edu.au/pages/resources.

### Sample size

To calibrate the decision thresholds for effectiveness, futility and superiority and evaluate the operating characteristics of the trial, we implemented pre-trial Monte Carlo simulations of hypothetical trials spanning a range of scenarios. Such methods are generally more informative than conventional power calculations as the frequency of type I and type II error is determined (by simulation) using a range of treatment effects of different size within the plausible range, including no difference (to evaluate type I error). In brief, we assumed a probability of the endpoint of 0.2 in the standard care arm, no drop-outs or missing data and analyses performed after every 200 enrolments. Estimated sample sizes varied by domain according to anticipated numbers recruited into that domain based on the time the domain will be open and domain specific eligibility. We evaluated scenarios in which no treatments in any domain were more or less effective than usual care (null scenario) and scenarios in which investigational treatments were associated with an OR of the endpoint of 1.1 (harmful), 0.8 (weak effect), 0.67 (moderate effect) and 0.5 (strong effect). For the anticoagulation domain, we evaluated a scenario where intermediate dosing was equivalent to prophylactic dosing, and therapeutic anticoagulation is the investigational treatment. For each scenario, we ran 10,000 simulations.

Table 2 provides a condensed summary of the simulation results where the pre-specified adaptive algorithm and decision thresholds are calibrated to ensure the design has a likelihood of type I error (false positive) that corresponds to a frequentist *P* value of less than 0.05. In the null scenario, an arm was falsely declared to be effective in < 5% of simulations and correctly declared as futile in > 50% of simulations. A moderately effective treatment was identified correctly as effective in at least 75% of simulations. In each domain, a strongly effective treatment was identified as effective and superior in > 85% of simulations after 1000 enrolments. Harmful treatments were identified as futile in > 80% of simulations.

**Table 2:** Summary of results of simulations indicating probabilities for arriving at platform conclusions (effectiveness or futility) in each domain with varying sample sizes and effect sizes. The probabilities for arriving at platform conclusions can be conceptualised as similar to the power of the study

	Antiviral domain		Anticoagulation domain		Antibody domain	
Treatment arms	2		3		2	
Estimated sample size	2000		3200		1600	
Odds ratio (effect)	Effective	Futile	Effective	Futile	Effective	Futile
1.10 (harmful)	0.01	0.89	0.00	0.80	0.01	0.83
1.00 (null)	0.03	0.67	0.03	0.53	0.03	0.58
0.80 (weak)	0.37	0.10	0.50	0.07	0.30	0.10
0.67 (moderate)	0.84	0.01	0.93	0.01	0.75	0.01
0.50 (strong)	1.00	0.00	1.00	0.00	0.99	0.00

### Biobanking

In order to maximise site participation and limit barriers to trial enrolment, the core protocol of ASCOT ADAPT does not involve any mandatory collection of biological samples. Nonetheless, there are many important research questions related both to specific therapeutic interventions and the cohort of participants enrolled in ASCOT ADAPT, and sites and participants can elect to contribute samples towards a COVID-19 biobank. Biological specimens collected include respiratory viral swabs, peripheral blood mononuclear cells, serum and plasma, collected at timepoints from baseline to 14 days after enrolment. A tiered consent from participants may allow samples or information to be used for any approved research projects and may also allow for host genomic studies to be undertaken with stored samples.

Biological specimens collected for ASCOT ADAPT are stored in participating biobank facilities, with a central ‘virtual biobank’ catalogue of samples and consent. Samples are collected, processed and stored according to central laboratory procedures. Access to samples for ethics-approved protocols is considered through a central biobank committee, where guiding principles for access are equity and maximising scientific value. Storage of specimens is provided by the ASCOT ADAPT study, with retrieval and transport costs funded by external researchers accessing specimens.

### Independent Data and Safety Monitoring Committee (DSMC)

The DSMC will review all unblinded serious adverse reactions at predetermined intervals during the study or as otherwise deemed appropriate by the DSMC. The DSMC is independent of the coordinating centre and investigators and will perform an ongoing review of predefined safety parameters, study outcomes and overall study conduct. A detailed charter between the DSMC and Study Management Committee outlining roles, responsibilities, processes of stopping rules, reporting and communication has been signed.

### Trial implementation and operation

The establishment of an international clinical trial during the COVID-19 pandemic has necessitated a number of special considerations in light of factors such as evolving infection control practices and travel restrictions affecting countries involved in ASCOT ADAPT. Participant information and consent forms, for example, would normally be signed by participants and then included in primary trial documentation; however, concerns regarding potentially infectious fomites meant this could not occur. Instead, ethics committees approved modifications to enrolment to allow telephone and videoconferencing to be used for consent, with researchers and witnesses completing documentation outside negative pressure facilities. Copies of signed documents were then provided to participants electronically, including complete details regarding confidentiality, use of data, withdrawal procedures, post-trial follow-up and information sharing and independent oversight. Individually identifiable data held in the central database is limited to unique trial identifiers, further supporting participant confidentiality.

Trial monitors for ASCOT ADAPT were also unable to travel to sites for evaluations due to these restrictions on movement and occupational health and safety requirements. A system of remote monitoring was established, with an independent team conducting review of documentation and processes, including routine review for the first patient at each site and randomly selected patients thereafter.

The trial in India is overseen by The George Institute for Global Health, Sydney, Australia and New Delhi, India. As of October 1, 2021, The World Health Organisation has recorded more than 30 million confirmed cases of COVID-19 and more than 400,000 deaths in India, making it the second highest country in the world in terms of cumulative reported cases. For the ASCOT ADAPT trial, we have also set up an India specific regional trial committee (chaired by Prof Vivekanand Jha, Executive Director of TGI, New Delhi). This committee has been meeting weekly. Each of the main domain specific working groups also includes Indian representation. There is community representation on the India specific trial committee. Therefore, we are confident that the study questions being asked are of priority to Indian patients and participating trial sites and feasible to address in India. We have ensured regular academic detailing with frequent online educational meetings and telephone conversations with site principal investigators and monthly newsletters. A 24 h a day, 7 days per week telephone advice hotline is also available to assist site recruitment. Given the geographically distributed nature of trial sites and infection control considerations, most monitoring has occurred with the use of Zoom and WhatsApp. Consent forms and patient medical records are sighted for source data verification by study monitors during remote site visits via Zoom.

At the time of writing, the anticoagulant domain is operational at 29 sites. The antiviral domain is active at 15 sites in Australia and NZ and awaiting regulatory approvals for Indian sites. There are 12 participating sites and more than 970 patients have been enrolled into the anticoagulant domain as of 1 October. Source data verification and monitoring are performed by the George Institute staff where feasible. When infection rates preclude site visits, then the data are verified online.

### Close out

At the time of completion of each study question, results will be released by the Trial Steering Committee and submitted for publication in peer-reviewed journal. At completion of the study, the monitor will ensure that there are plans in place for the long-term storage of all the relevant data and source documentation (for 15 years). The study drug will be reconciled and then destroyed according to local standard procedures.

### Barriers and challenges

Inherent in the concept of an adaptive trial is the evolution of interventions and domains under investigation. The ASCOT ADAPT study was established with the expectation that data gathered from within the trial would inform ongoing randomisation and also that externally generated information from other trials could be considered by the DSMC and Trial Steering Committee. Since initiation of the original ASCOT study, several potential therapeutic interventions have been removed, based largely on data released from other studies. These interventions include hydroxychloroquine, lopinavir/ritonavir and interferon-beta, which were included in early protocols but did not have patients randomised, and convalescent plasma, which had patients randomised prior to external evidence indicating a lack of effect in comparable patient cohorts. The anticoagulation domain has also been amended to cease enrolment to aspirin-containing arms, following a lack of evident benefit. For both convalescent plasma and aspirin, individual participant data is available for analysis and for contribution to meta-analyses, in order to maximise the scientific benefit arising from participant enrolment in these arms and domains. While such protocol amendments are not without effort, it is considerably easier to accomplish than to establish new trial protocols, and highlights the flexibility of the ASCOT ADAPT trial to respond to changing evidence and optimise value for participants and the community.

The ASCOT ADAPT study does not exclude potential participants on the basis of breastfeeding or pregnancy, although some domains or interventions may contraindicate enrolment. While many interventional studies routinely exclude pregnant or breastfeeding women, we consider it an important matter of equity to allow such informed participation where no clear contraindication exists. We did, however, experience significant obstacles with regards to obtaining insurance for pregnant trial participants, and hope that as more clinical trials support access to full participation, such trial logistic processes will become more straightforward.

Initial core funding for ASCOT ADAPT came primarily from a range of philanthropic donors. An adaptive platform such as ASCOT ADAPT will have most value if it is maintained over a prolonged period, however, and ensuring continuity of funding for platforms as well as specific interventions will be required.

Finally, the challenges of multi-jurisdictional research ethics and governance are well-recognised. Our experience has been that human research ethics committees have been extremely supportive of the ASCOT ADAPT trial design and implementation and have been receptive to innovative methodology with regards to aspects such as consent and source documentation in light of infection control considerations. We have had most significant hurdles in trial initiation arise from institutional governance processes, which have frequently been protracted and appeared poorly suited to collaborative research programs under pandemic conditions. In considering readiness for future pandemic research or preparedness for other rapidly emerging health conditions, pre-existing cross-institutional agreements would provide substantial benefits, as would agreement to rely on standard templates for common institutional agreements. We have made further recommendations to facilitate coordinated and timely operationalisation of clinical trials during a pandemic in the Australian context [35].

## Discussion

The rapidly changing conditions associated with the COVID-19 pandemic have presented a variety of challenges for optimal management and research. This has included changing geographical and demographic patterns of disease, with treatment centres at different times experiencing either an absence or overwhelming burden of illness, and shifting evidence for a large number of potential therapeutic options. While some of these barriers have limited timely progress, they also underscore the value of a platform such as ASCOT ADAPT, which aims to be pragmatic in its design, flexible with regards to its interventions, and able to be in place across a large number of sites in different global regions in anticipation of future pandemic trends.

In many countries, large-scale vaccination programs have been presented as key to ending the COVID-19 pandemic. While the speedy development and testing of effective vaccines has been a magnificent accomplishment, much of the world has yet to access vaccines on a large-scale basis, some vulnerable individuals may be ineligible or contraindicated for vaccination, waning immunity and the emergence of variants with potential for vaccine escape is anticipated. Even as vaccine programs do expand globally, it remains the case that a proportion of vaccinated people exposed to SARS-CoV-2 will continue to develop clinically significant COVID-19, and there will be continued need for effective therapeutic agents with established safety profiles. Patients who are hospitalised are at high risk for progression to intensive care admission and/or death, and the ASCOT ADAPT trial focus on preventing these outcomes will remain critical.

Finally, while ASCOT ADAPT has been established to focus on COVID-19 therapeutics, it has also established an adaptive international network of infectious diseases and respiratory clinicians managing hospitalised patients with non-critical respiratory infection. Platform trials such as REMAP-CAP have used such networks for a range of associated interventional studies in ICU patients, but there has not been a similar platform previously available for considering optimalisation of therapy for less critically unwell patients. As the COVID-19 pandemic evolves over time, ASCOT ADAPT will consider how the longevity of the platform may be extended and potentially used to support gathering evidence to improve management across a wider range of conditions than COVID-19 alone.

## Supplementary Information

**Additional file 1.** ASCOT ADAPT Statistical Analysis Appendix.

## Data Availability

The authors retain full control over study dataset, with no contractual limitations on access. Following completion of study domains, publication of results will be sought, including data sharing to support individual participant meta-analysis and other joint works. Authorship of resulting publications will follow ICMJE guidelines, with no use of professional writers.
