# Supplementary material for: ASCOT ADAPT study of COVID-19 therapeutics in hospitalised patients: an international multicentre adaptive platform trial
Source: Trials. 2022 Dec 14;23:1014. doi: 10.1186/s13063-022-06929-y (PMC9747535; doi:10.1186/s13063-022-06929-y)
Supplement: Supplementary file 1 — Additional file 1. ASCOT ADAPT Statistical Analysis Appendix. [file 13063_2022_6929_MOESM1_ESM.pdf]

ASCOT

AustralaSian COVID-19 Trial

ASCOT ADAPT Statistical Analysis Appendix

Nov 2021

SAA Version: 2.2

Based on core protocol version: 5.0

Contents

Version History 5

1 Introduction and Purpose 6

2 Structure of Trial 7

2.1 Target Population . . . 7
2.2 Treatment Domains . . . 7
2.3 Regimens . . . 8
2.4 Standard of Care . . . 8
2.5 Subgroups . . . 8
2.6 Randomisation . . . 9

3 Endpoints and Estimands 10

3.1 Primary Outcome . . . 10
3.2 Secondary Outcomes . . . 10
3.3 Covariates . . . 10
3.4 Estimands . . . 10

4 Statistical Modelling 11

4.1 Analysis Population . . . 11
4.2 Primary Model . . . 11
4.3 Models for Secondary Outcomes . . . 12
4.3.1 Binary Outcomes . . . 13
4.3.2 Ordinal Outcomes . . . 13
4.3.3 Time-to-event Outcomes . . . 13
4.4 Primary Model Priors . . . 14
4.4.1 Treatments . . . 14
4.4.2 Treatment interactions . . . 14
4.4.3 Regions . . . 14
4.4.4 Sites . . . 14
4.4.5 Temporal Cohorts . . . 15
4.4.6 Age Parameters . . . 15
4.4.7 Other Covariates . . . 15
4.4.8 Intercept terms for ordinal models . . . 15

4.5 Subgroup Analyses . . . 16
4.6 Ineligible or Unavailable . . . 16
4.7 Missing Data . . . 17
4.8 Sensitivity Analyses . . . 17
4.9 Model Deviations . . . 17

5 Statistical Quantities **18**
5.1 Best Regimen . . . 18
5.2 Best Treatment (treatment in best regimen) . . . 18
5.3 Treatment Contrasts . . . 19

6 Decision processes **20**
6.1 Platform conclusion procedure . . . 20

7 Trial Decision Criteria **21**
7.1 Effectiveness . . . 21
7.2 Futility . . . 21
7.3 Superiority . . . 21
7.4 Inferiority . . . 22

8 Trial Adaptations **23**
8.1 Sequential Analyses . . . 23
8.2 Actions Arising from Platform Conclusions . . . 23
8.2.1 Superiority . . . 23
8.2.2 Inferiority . . . 23
8.2.3 Effectiveness . . . 24
8.2.4 Futility . . . 24
8.3 Response-Adaptive Randomisation . . . 24
8.4 Adding Interventions . . . 25

9 Reporting **26**
9.1 Blinding . . . 26
9.2 Model Parameter Summaries . . . 26
9.3 Participant Progression . . . 26
9.4 Participant Characteristics . . . 26
9.5 Protocol Adherence . . . 27

9.6 Graphical and Descriptive Summaries 27
9.7 Missing Data 28

References **29**

Version History

Version 2.2 - Nov 2021

- Authors: Mark Jones

1. Minor typo edits
2. Simplified modelled notation removing interaction
3. Removed placeholders for delayed reveal and evolution of soc.

Version 2.1 - Aug 2021

- Authors: Mark Jones

1. Clarified purpose and limitations
2. Revised structure, migrated content from SAP
3. Removed duplication taken from other documents

Version 2.0 - Oct 2020

- Authors: James Totterdell

1. Revise RAR rules to enforce minimum of 1/3 to each intervention in a domain of only two interventions

Version 1.0 - Aug 2020

- Authors: James Totterdell

1. Statistical analysis plan ASCOT ADPAT

1 Introduction and Purpose

This document presents the general framework for the statistical design and analysis plan for ASCOT-ADAPT. It is an entry point for analysts and others to gain familiarity with the technical aspects of the trial. Additionally, the document is intended to provide sufficient detail to satisfy governance, oversight and/or external bodies that the trial has a sound theoretical basis and has adequately pre-specified the analyses and related matters.

The following documents were reviewed when preparing this document:

- Core protocol for ASCOT-ADAPT
- Domain-specific appendices to the core protocol
- CRFs are reviewed at their current versions if applicable.

Statisticians working on analyses for the platform must read this document in conjunction with the other statistical documentation to get a complete understanding of the analytical approach. While the Statistical Appendix is intended to undergoe relatively little change over time, the Implementation Guide is specific to the current state and structure of the trial and is expected to change.

Our implementation of the analytical documentation hierarchy aims to minimise duplication at the cost of some cross-referencing between documents. Concepts and topics introduced in higher level documents can be expanded upon in lower level documents (such as the Implementation Guide), but as a rule, duplicating content from other documents is avoided.

Specifically, analysts must read this document in conjunction with the Implementation Guide document, the Simulation Appendix and the consolidated Statistical Glossary and Abbreviations. We also note that domain specific variations might be included into the statistical considerations sections of the domain specific appendices.

We have purposely kept the level of detail to a minimum in order to constrain the size and simplify documentation management processes. Additionally, in order to provide a stable and relevant characterisation of the analytical approach over the duration of the trial, the content of this document avoids referencing specific treatment interventions. In brief, the contents of this document are as follows. Section 2 outlines the general trial structure, Section 3 defines and provides some brief discussion on the trial outcome measures of interest, Section 4 introduces the statistical models and priors, Section 5 presents the model quantities which will be used at analyses to inform trial decisions and adaptations, Section 6 outlines the platform conclusion procedure, Section 7 describes the quantities used to make operating decisions for the trial, Section 8 outlines trial adaptations and Section 9 discusses trial reporting.

This document was written and reviewed by those authors detailed in the Version History section. All contributors were blinded to treatment allocations and treatment-related study results at the time of their contributions. The versions of the statistical documentation applicable at the time of each interim analysis are bundled under the github release directory.

2 Structure of Trial

2.1 Target Population

The inclusion and exclusion criteria of the target population are introduced and discussed in the Core Protocol.

2.2 Treatment Domains

A treatment domain comprises a collection of competing interventions within a common clinical modality.

An intervention might be a unique compound, such as Tocilizumab, or the combination of multiple compounds administered simultaneously, such as Tocilizumab plus Kaletra. Alternatively, there may be multiple interventions within a domain that are the same compound, but administered at different dosages.

For generalisability, this document discusses the analyses in terms of generic domains without reference to the specifics, which are primarily detailed in the core protocol and domain specific appendicies.

While the number of domains will change as the trial evolves, here we only consider three domains. These domains are denoted by capital letters, A, B, C, and a generic domain will be represented by d. Within each domain there will be a number of distinct interventions denoted by subscripts, $d_1, d_2, ...d_{K_d}$ where 1 generally indicates no treatment or standard of care within that domain. K_d denotes the total number of interventions available in the domain over the course of the trial and K_d' will be used to indicate the number of treatments that are currently open to enrolment. We also include a special arm d_0 as a way to denote 'not randomised.'

For a participant to be randomised, there must be two or more interventions in one or more domains available to them at the enrolling site. However, there are several scenarios under which the full set of regimens are not available to a participant. These scenarios relate to site-level and/or participant-level characterisitics, for example:

- one or more domains are not open for enrolment at the site where the participant is to be enrolled
- one or more treatments within one or more domains are not available for assignment at the site where the participant is enrolled
- the participant is not eligible for one or more domains
- the participant is not eligible for one or more treatments within one or more domains

Additionally, consideration must be given to the temporal evolution of the trial whereby new domains and/or interventions are added at some time after the start of the trial. As none of the existing participants were randomised to the new domains, they will not contribute to the evaluation of relative effects within those domains. More concretely, these participants will not contribute as members of the 'control' arm within the added domains as they will be given the 'not randomised' d_0 assignment.

If only a subset of domains are available to a participant, then they can be randomised to the available domains. As these participants are excluded from contributing to the relative comparisons within the other domains, their individual likelihood will include a 'not randomised' parameter for each excluded domain. Clearly, while a 'not randomised' arm d_0 is accommodated within one or more of the other domains, participants should not be thought of as having been randomised to these arms.

If only a subset of interventions are available within a domain, then the participant can still be randomised to this domain. However, the allocation probabilities must be re-weighted for this participant prior to randomisation. Typically this might be achieved by dividing the allocation probabilities for the available arms by the sum of these probabilities.

Further detail on accounting for these scenarios can be found in the Ineligible or Unavailable section and in the Implementation Guide document.

As the trial progresses, domains may be added, new interventions may be added to an existing domain and existing interventions may be permanently halted (e.g. a futile arm) or suspended (e.g. suspend standard of care when no investigational arms are open to enrolment). If there are no arms open to enrolment in a domain, then it is considered closed. The maximum number of arms that are available within a domain and across the whole trial are informed by simulation to ensure acceptable trial performance characteristics.

2.3 Regimens

Each trial participant is randomly allocated to a regimen, which comprises a collection of interventions, one selected from each treatment domain. Assuming that every intervention from each domain may be given in combination with all interventions from every other domain, the number of distinct regimens is equal to $K_A \times K_B \times K_C$. A treatment regimen may be denoted by an index $j = 1, 2, ..., K_A K_B K_C$ or by a string indicating the component interventions. For example, the regimen composed of treatment 1 from domain A, treatment 2 from domain B and treatment 0 from domain C (not randomised to domain C) may be represented by $A_1 B_2 C_0$. For any particular regimen j, the notation $d(j)$ will refer to the intervention from domain d which forms part of the regimen j, such as $\{A(j) = A_1, B(j) = B_2, C(j) = C_0\}$ for the previous example regimen.

2.4 Standard of Care

Standard of care is described in the Core Protocol although this description may be extended/varied in domain specific appendicies. Data will be collected on any agents used as standard and adjusted for in the statistical model where deemed relevant, see Covariates and the Implementation Guide.

Note that as participants are randomised to regimens, e.g. $A_1 B_2 C_0$, they may simultaneously contribute to the estimation of parameters associated with the standard of care in some domains and investigational arms in other domains.

2.5 Subgroups

Treatment effect heterogeneity will be explored for subgroups defined by the following variables as measured at baseline across all domains:

- country/region
- days since symptom onset ≤ 7 days or > 7 days
- < 60 years of age or ≥ 60 years of age
- receipt of corticosteroid
- receipt of remdesivir
- receipt of other agent intended to be an antiviral agent against SARS-CoV-2
- participants receiving ACE inhibitor/ATII blocker therapy at the time of presentation
- required supplemental oxygen at time of randomisation or oxygen saturation less than 94% at room air

Other domain-specific subgroup analyses may be specified in the domain specific appendices. Any other subgroup analyses will be post-hoc and reported as such.

2.6 Randomisation

Initially, all interventions within a domain (and therefore all regimens) will be allocated with equal probability. Participants will only be randomised to regimens comprising interventions for which they are eligible and interventions that are available at the time of enrolment.

Following an analysis, some interventions in a domain might be (permanently) dropped from the platform, or new interventions added. Interventions in a domain that have a non-zero randomisation probability (including standard of care) will be referred to as intervention arms that are open for enrolment or *live interventions*.

Response adaptive randomisation will be used to update the allocation ratios for the live intervention arms following each scheduled analysis. For domains with more than 2 actively allocated interventions, the standard of care option (if still active) will have a fixed allocation of $1/K_d'$ where K_d' is the number of actively allocated interventions in the domain. If a domain has only 2 interventions (one of which is standard of care), then the minimum allocation probability is $1/3$. If the SOC has been replaced then that arm receives fixed allocation as above from the time it replaces the SOC.

Further detail on the specific randomisation algorithm used is provided in the Response-Adaptive Randomisation section.

3 Endpoints and Estimands

3.1 Primary Outcome

The primary outcome is discussed in the Core Protocol. However, for the purposes of this document, the primary outcome is a dichotomous variable with death from any cause or requirement of new intensive respiratory support (invasive or non-invasive ventilation) or vasopressor/inotropic support in the 28 days after randomisation coded as one.

3.2 Secondary Outcomes

The secondary outcomes are introduced in the Core Protocol and include dichotomous, ordinal and positive discrete random variables.

3.3 Covariates

Baseline covariates which will be part of the primary analyses include:

- country/region
- site
- epoch of enrolment
- age < 60 or ≥ 60 years

The Implementation Guide discusses the way these terms are parameterised.

3.4 Estimands

The primary estimand will be the log-odds of the primary endpoint for each treatment relative to standard of care at the planned endpoint of 28 days after randomisation for all randomised participants irrespective of post-randomisation events, see Analysis Population and Missing Data. The primary estimand is applied in the Sequential Analyses.

A secondary estimand will be the log-odds of the primary outcome for each treatment relative to standard of care at the planned endpoint of 28 days after randomisation for participants without protocol deviations. Primary endpoint data collected after protocol deviations have occurred will not be included in this secondary analysis.

4 Statistical Modelling

Inferences in the trial will be based on Bayesian methods. The models used will account for the trial implementation by adjusting for variation in outcomes by region (country), site, time since trial commencement and age. In general, the primary model will estimate treatment effects assuming no interaction between treatments across different domains. However, specific combinations may consider interactions and these will be detailed in the Implementation Guide. Secondary models will also investigate interaction effects across treatment domains and treatment effect heterogeneity by subgroup. All model posterior distributions and derived quantities will be estimated using Markov chain Monte Carlo draws from the joint posterior density.

Throughout of this section, the following notation will be used:

- $r = 1, ..., R$ denotes regions.
- $s = 1, ..., S_r$ denotes sites within a region.
- $t = 1, ..., T$ denotes participant cohort grouped according to time of enrolment
- d_k denotes treatment k within domain $d \in \mathcal{D}$, where $\mathcal{D}$ denotes the set of domains.

4.1 Analysis Population

The primary analysis population will inform the primary estimand and includes all participants who were randomised to at least one of the interventions and have passed the primary endpoint of 28 days after randomisation with their primary endpoint status either known or known to be missing.

This primary analysis population will be used for all core outcomes and will follow the intention-to-treat (ITT) principle. All randomised patients will be included and analysed according to the regimen to which they were initially allocated irrespective of any deviations from this regimen or any other protocol deviations.

Participants that have reached follow up, but for whom information has not yet been gathered will be treated as missing until the data has been entered. This assumes that we believe that data entry nor retrieving participant outcome status is differential across treatment groups. Participants who have been randomised, but have not yet reached the primary endpoint, will be excluded.

A secondary analysis population will include all participants who are randomised to at least one of the interventions. However, this analysis set will follow the per-protocol (PP) definition with randomised patients included in the analysis only if no protocol deviations occurred prior to the endpoint.

Intervention availability may vary over time and both domain and eligibility criteria for interventions may also vary. Therefore, a number of analysis populations will be of interest to inform sensitivity and additional analyses complementing the results from the primary set of analyses.

The final analysis (for the platform conclusion) will only use data for those participants who were enrolled into the platform prior to the platform conclusion.

Further detail on analysis sets can be found in the Primary Model Document.

4.2 Primary Model

For a participant i enrolled in the study the notation $r(i) = 1, ..., R$ will be used to indicate the region to which that participant belongs, similarly for site, $s(i)$ and cohort $t(i)$.

The primary outcome will be modelled by logistic regression for a participant $i = 1, 2, 3, \dots, n$ as follows

$$\pi_i = \text{logit}^{-1}(\eta_i)$$
$$\eta_i = \beta_0 + \sum_d x_{d(i)}^\mathsf{T} \beta_d + \rho_{r(i)} + \xi_{r(i),s(i)} + \tau_{t(i)} + z_i^\mathsf{T} \alpha$$

where

- π_i is the probability of response for participant i
- η_i is the log-odds of response for participant i
- β_0 represents the baseline log-odds of response in the reference population
- β_d is a vector of parameters for each domain reflecting the effect of each treatment which depends upon the domain design matrix X_d of which $x_{d(i)}^\mathsf{T}$ is a row
- ρ_r is the change in log-odds associated with region r
- $\xi_{r,s}$ is the change in log-odds associated with site s nested within region r
- τ_t is the change in baseline response specific to participants recruited during epoch t
- α represents other covariates specified for model inclusion (see Covariates, but also includes intervention specific ineligibilities)

Notes:

- Interactions between treatments in different domains may be investigated as part of an extended model where deemed relevant.
- Only two-way interactions will be considered.
- The design matrices may include interactions for combinations of interventions within a domain. For example if intervention d_3 was the combination of d_1 and d_2 given together as opposed to each alone then β_{d3} would denote an interaction term coefficient.
- Interactions may only be of interest for a subset of domains or interventions with the rest having any redundant parameters fixed to zero.

Finally, at times (see Best Regimen) it might be more useful to consider the model in terms of the the response under each regimen $j = 1, 2, ..., K_A K_B K_C$ i.e.

$$\eta_j = \beta_0 + \sum_d x_{d(j)}^\mathsf{T} \beta_d$$
$$\pi_j = \text{logit}^{-1}(\eta_j)$$

where $d(j)$ returns the treatment from domain d which is used in regimen j.

4.3 Models for Secondary Outcomes

The analysis model of each secondary outcome will use a similar model structure (linear predictor) as that used for the primary outcome. Binary variables will be analysed by logistic regression, ordinal outcomes by cumulative logistic regression, and discrete time-to-event outcomes by continuation ratio logistic regression

4.3.1 Binary Outcomes

An independent Bayesian logistic regression model will be used for each binary outcome. The outcome will be coded so that the odds-ratios have a logical direction for implying a treatment benefit. For example, if we are considering death as the response, then the coding will be such that an odds-ratio < 1 will imply a reduction in the odds of death and thus treatment benefit. The model form will be

$$\text{logit}\left[\text{Pr}(y_i = 1)\right] = \eta_i, \quad i = 1, ..., n$$

for participant i with outcome y_i, and linear predictor η_i. The linear predictor, η_i, will take a similar form to the primary analysis model and the model will use the same priors. Sensitivity analyses may vary the form of the linear predictor.

Referencing the Core Protocol, the binary secondary outcomes are:

- all-cause mortality at 28 days after randomisation
- dichotomous comparison of a subjective measure of shortness of breath at 28 days after randomisation

4.3.2 Ordinal Outcomes

An independent Bayesian cumulative logistic regression model will be used for each ordinal outcome. Similar to Binary Outcomes, the outcome will be coded so that the odds-ratios have a logical direction to imply treatment benefit. The model form will be

$$\text{logit}\left[\text{Pr}(y_i > j)\right] = \alpha_j + \eta_i, \quad j = 1, ..., J-1, \quad i = 1, ..., n.$$

for participant i, with outcome category y_i, category specific intercept α_j, and linear predictor η_i. The linear predictor, η_i, will take the same form as in the primary analysis model and the model will use the same priors on parameters in the linear predictor. The model will assume proportional odds effect of each treatment across outcomes, i.e. η is assumed to be constant across categories. Sensitivity analyses will vary the form of the linear predictor and assume a more informative prior on the category specific probabilities.

Referencing the Core Protocol, the ordinal secondary outcomes are:

- WHO 8-point ordinal outcome scale at day 28 after randomisation
- Days alive and free of hospital by 28 days after randomisation
- Days alive and free of invasive or non-invasive ventilation by 28 days after randomisation
- Ordinal comparison of the modified Medical Research Council (mMRC) breathlessness scale.

4.3.3 Time-to-event Outcomes

An independent discrete-time-to-event proportional continuation ratio logistic model (Allison, 1982; Cox, 1982) will be used for each time-to-event outcome *measured in days*. Similar to Binary Outcomes, the outcome will be coded so that the odds-ratios have a logical direction to imply treatment benefit. Outcomes are censored as specified in the outcome definition (e.g. at day 28). The model will assume proportional conditional odds ratios of each intervention across all times. The model form will be

$$\text{logit}\left[\text{Pr}(y_i = u | y_i \geq u)\right] = \alpha_u + \eta_i, \quad u = 1, ..., U$$

where u denotes discrete time (e.g. days), α_u is the time-varying intercept and η_i the participant specific linear predictor. The linear predictor, η_i, will take the form as in the primary analysis model and the model will use the same priors on parameters in the linear predictor. Under this model, the hazard is defined as a conditional probability and the intercepts can be interpreted as the logit (yes, *logit*, not *log*) of the baseline hazard and the linear predictor represents the additive effect of the covariates on the logit of the hazard. Sensitivity analyses will vary the form of the linear predictor and assume a more informative prior on the baseline log continuation ratios (e.g. smoothness).

Referencing the Core Protocol, the time-to-event secondary outcomes are:

- Time to clinical recovery (in days) during the first 28 days after enrolment

4.4 Primary Model Priors

4.4.1 Treatments

The baseline-response (intercept term) β_0, and the treatment effects β_d are given the following priors

$$\beta_0 \sim \text{Normal}(0, 2.5^2)$$
$$\beta_{dk} \overset{\text{iid}}{\sim} \text{Normal}(0, 1), \quad k = 0, 1, ..., d_{K_d}, \ d = 1, 2, 3, ...$$

The treatment effect parameter β_d consists of a reference treatment, β_{d1}, and will also include a term for not being randomised, β_{d0}, to account for ineligibility and unavailability. For example, suppose at trial commencement two domains A and B are open and later in the trial a third domain C is opened. Participants who entered the trial prior to domain C opening were not randomised in that domain. Given that domain C was unavailable to these participants they would only contribute to β_{C0} (the not randomised parameter) in domain C.

4.4.2 Treatment interactions

Treatment interactions may arise within a domain or across domains. The treatment effect parameters for interactions are specified on a case by case basis in the Implementation Guide. However, as a general rule an informative prior on no interaction effect is specified.

4.4.3 Regions

Region $r = 1$ will be the reference region and all other regions $r = 2, ..., R$ will have prior

$$\rho_1 = 0$$
$$\rho_r \overset{\text{iid}}{\sim} N(0, 1), \quad r = 2, ..., R.$$

4.4.4 Sites

Sites are nested within region and will be treated as exchangeable within region with priors

$$\xi_{rs} \overset{\text{iid}}{\sim} \text{Normal}\left(0, \sigma^2_{\xi_r}\right), \quad s = 1, ..., S_r \quad r = 1, ..., R.$$
$$\sigma_{\xi_r} \overset{\text{iid}}{\sim} \text{Half-}t(3, 1),$$

The mean of zero implies that on average the sites in region r have expected baseline-response $\beta_0 + \rho_r$.

4.4.5 Temporal Cohorts

There is potential for both background care and circulating virus strains to show structural variation (i.e. not just random variation due to noise) as the trial progresses. Participants recruited closer together in time are expected to have a more similar experience than those recruited further apart in time. The use of response-adaptive randomisation means that allocation ratios to interventions will be updated and therefore intervention effects may be confounded by these temporal changes.

To account for temporal trends, participant are grouped into sequential cohorts relative to the most recently randomised participant included in the analysis and thus relating to a particular epoch in the trial. Epochs span 4 week windows and participants are included in a single cohort, although cohorts may be pooled with the next most recent one if there is insufficient data ($<$ 5 randomised participants).

The prior for the models time component will be a random-walk

$$\begin{aligned}
\tau_1 &= 0 \\
\tau_t &= \tau_{t-1} + \sigma_\tau \epsilon_t \\
\epsilon_t &\overset{\text{iid}}{\sim} N(0,1), \quad t = 2, ..., T \\
\sigma_\tau &\sim \text{Half-}t(3,1).
\end{aligned}$$

where t = 1 implies that the participant was randomised within 4 weeks of the most recent participant, t = 2 that they were randomised more than 4 weeks past but within 8 weeks, etc. This prior enforces some smoothing of the baseline response across cohorts expecting only small variations between cohorts in temporal proximity.

4.4.6 Age Parameters

Age is dichotomised as less than 60 years of age (reference category), or greater than or equal to 60 years of age. The parameter priors will be

$$\begin{aligned}
\alpha_0 &= 0 \\
\alpha_1 &\sim \text{Normal}(0, 2.5^2).
\end{aligned}$$

4.4.7 Other Covariates

Nominally, other covariates parameters will assume a default prior of

$$\alpha \overset{\text{iid}}{\sim} N(0, 2.5^2)$$

with variations specified in the Implementation Guide.

4.4.8 Intercept terms for ordinal models

The prior for the intercept terms for ordinal models will be implied from a uniform Dirichlet prior on the category specific probabilities.

4.5 Subgroup Analyses

For subgroups, the primary analysis model (ignoring the potential interaction components for simplicity of presentation) will be extended to allow for varying treatment effects by subgroup. For example, for region the model would be extended via

$$\eta_i = \beta_0 + \sum_d x_{d(i)}^{\mathsf{T}} \beta_d + \sum_d x_{d(i)}^{\mathsf{T}} \rho_{d,r(i)} + \rho_{0,r(i)} + \zeta_{s(i)} + \tau_{t(i)} + z_i^\top \alpha$$

$$\rho_r | \Omega_\rho \overset{\text{iid}}{\sim} N(0, \Omega_\rho),\ r = 1, \dots, R \quad \Omega_\rho \sim p(\Omega_\rho)$$

where $p(\Omega_r)$ is the prior on the covariance of the region treatment effects. We specify the priors on the marginal standard deviations and correlation separately (Joe, 2006; Lewandowski et al., 2009; Tokuda et al., 2011)

$$\begin{aligned} \Omega_\rho &= \text{diag}(\omega)\Lambda\text{diag}(\omega) \\ \omega_l &\sim \text{Half-}t(3, 1) \\ \Lambda &\sim \text{LKJ}(1). \end{aligned}$$

4.6 Ineligible or Unavailable

At the time of enrolment, a participant may be ineligible for one or more domains. If a participant is ineligible for a given domain then that participant will not be randomised to any intervention for that domain. However, the participant will be included in the primary analysis as long as they are eligible for at least one other domain. A covariate will indicate ineligibility for each applicable domain to account for possible association between participant factors determining domain ineligibility and the primary outcome.

Alternatively, a participant may be eligible for all domains, but ineligible for domains-specific interventions as follows:

- If a participant is ineligible for all the interventions that are open for enrolment in the domain then they will be treated as ineligible for the domain itself (even if they happen to be eligible for an inactive intervention within the domain).
- If a participant is only eligible for one of the interventions open for enrolment, then the participant may receive it, however, they will be treated as ineligible for the domain itself.
- If a participant is eligible for at least two actively allocated interventions, the participant will be randomised amongst those eligible interventions and treated as eligible for the domain but ineligible for the other interventions in the domain.

When applicable, the participant will be included in the primary analysis and a covariate indicating their intervention specific ineligibility will be included to account for possible associations between participant factors determining ineligibility for a particular intervention and the outcome. Each intervention will have it's own ineligibility effect as required. The covariate vector, e_i, which is used to indicate intervention ineligibility will be included in the primary model and the associated parameters will use the prior

$$\xi \sim N(0, 10^2).$$

The intervention specific eligibilities are coded as standalone co-variates introduced as needed separate from the domain specific ones. For example, if there was a specific ineligibility for treatment $A2$ only, then a new variable e_{A2}, say, would be introduced indicating that a participant was ineligible for $A2$, but could still be randomised to another intervention in domain A, allowing for a different baseline among such participants. That participant may still be eligible for $A1$, $A3$, $A4$ and is therefore still randomised to the domain. While the domain coding would stay the same, if $e_{A2} = 1$,

then the participant cannot have received $A2$, meaning they aren't directly comparable to those participants who could have received $A2$. Domain ineligibility takes priority over intervention ineligibility, e.g. if ineligible for the domain, then it doesn't matter that they were also ineligible for $A2$.

The assumption being made in the above is that ineligibility for any domain or treatment, and also unavailability of any domain or treatment, does not modify treatment response to the interventions in the other domains.

More detail on the parameterisation can be found in the Implementation Guide document.

4.7 Missing Data

In the primary analysis, missing primary outcome data will not be imputed and participants without primary outcome data will be excluded from the sequential analyses. For example, participants that have reached the 28 day endpoint, but for whom information has not yet been gathered, will be treated as missing.

If the randomisation assignment is missing the patient will be assumed to be ineligible for that domain. If a participants eligibility is unknown, they will be assumed to be ineligible for that domain or intervention.

Missing covariate information may be imputed based on other available data (e.g. missing region, site or time of enrolment). Alternatively, the category mean will be used as the imputed value.

4.8 Sensitivity Analyses

Sensitivity analyses may include applying the same model to a different analysis population or varying the primary model. In particular, the following sensitivity analyses will be explored:

- the per-protocol analysis.
- analysis based solely on contemporaneous controls, defined as enrolments in the last 3 months.
- separate models fit to domain eligible subsets:
  - for each domain analyses will restrict to only those participants who were eligible for the domain
  - for each domain analyses will restrict to only participants who were eligible for all interventions in the domain
- sensitivity of the results to the choice of priors: allowing priors to be less or more informative than those specified in this document.
- method of handling missing primary outcome data, e.g. complete-case analysis, worst-case, or best-case scenarios.
- varying the assumption made for the primary endpoint regarding participants who discharged against medical advice.

4.9 Model Deviations

The primary analysis model will be assessed for adequacy. Additional models (either simpler or more complex) may be investigated as part of checks of sensitivity, stability, and model fit. If any issues or concerns arise (for example, strong evidence of interactions across treatment domains), all changes or updates to the specified primary model will be documented and reported.

5 Statistical Quantities

Certain quantities derived from the model parameter posterior densities will be used to inform the response adaptive randomisation and trial decisions. Posterior quantities of particular interest are defined here.

5.1 Best Regimen

In the Primary Model section, η_j was defined as the log-odds of response under a given regimen. Defining $j^\star = \text{argmin}_j(\eta_j)$ to be the **regimen** which minimises the log-odds of response, the probability that regimen j is the best regimen (in terms of minimising the log-odds of response) is

$$\phi_j = \mathbb{P}[\text{regimen } j \text{ is best}] = \mathbb{P}[j^\star = j] = \mathbb{P}[\eta_j < \eta_l, \forall l \neq j], \quad j = 1, ..., K_A K_B K_C.$$

5.2 Best Treatment (treatment in best regimen)

Recalling that for any particular regimen j, the notation $d(j)$ refers to the intervention from domain d which forms part of the regimen j (see Regimens), we define the probability that a treatment within a domain d is in the best regimen $j^\star$ by

$$\varphi_{dk} = \mathbb{P}[\text{treatment } d_k \text{ is in best regimen}] = \mathbb{P}[d(j^\star) = k], \quad k = 1, ..., K_d \quad .$$

Since each regimen contains only one intervention from each domain (which may be no intervention) the probabilities satisfy $\Sigma_{k=1}^{K_d} \varphi_{dk} = 1$ for each domain.

In the absence of interactions across domains, this is equivalent to

$$\varphi_{dk} = \mathbb{P}[\text{treatment } d_k \text{ is best in domain}] = \mathbb{P}\left[\text{argmin}_{l=1,...,K_d}(x_{d(l)}^\intercal \beta_d) = k\right], \quad k = 1, ..., K_d$$

where $x_{d(l)}^\intercal$ is a row from the domain design matrix corresponding to a particular treatment arm (including SOC if present) and therefore $x_{d(l)}^\intercal \beta_d$ is the contribution to the linear predictor for treatment l in domain d. If the best option from domain d is the one that reduces the log-odds by the largest amount and β_d is known then the best treatment arm is clearly

$$k = \text{argmin}_{l=1,...,K_d}(x_{d(l)}^\intercal \beta_d)$$

However, β_d is random, therefore our interest rests in

$$\mathbb{P}\left[\text{argmin}_{l=1,...,K_d}(x_{d(l)}^\intercal \beta_d) = k\right], \quad k = 1, ..., K_d$$

i.e. the probability that the treatment arm is best, as above.

If an intervention k in domain d has low probability of being the best then the intervention may be dropped. If one intervention has high probability of being the best then all other interventions in the domain may be dropped.

5.3 Treatment Contrasts

We define the probability that an intervention has a lower log-odds of the outcome than another intervention in the same domain by

$$\psi^d_{kl}(\Delta) = \mathbb{P}[\text{intervention } d_k \text{ better than intervention } d_l] = \mathbb{P}\left[x^{\mathsf{T}}_{d(k)}\beta_d < x^{\mathsf{T}}_{d(l)}\beta_d + \Delta\right], \\ k, l \in \{1, ..., K_d\}.$$

where Δ is a reference relative treatment effect.

For example:

- The probability that treatment $k > 1$ in domain d is effective (better than standard of care, $k = 1$) is $\psi^d_{k1}(0)$.
- The probability that treatment k is futile (reduces the log-odds of response by no more than $-\log(1.1)$) compared to no treatment is $\psi^d_{k1}(-\log(1.1))$.
- The probability that treatment k is non-inferior to a treatment l (reduces the log-odds of response by no less than $\log(1.1)$) is $\psi^d_{kl}(\log(1.1))$.

The concepts of effectiveness, futility and non-inferiority are expanded upon in the Trial Decision Criteria section.

6 Decision processes

For adaptations internal to the trial, predefined rules are evaluated based on statistical decision quantities derived from the primary model and pre-specified thresholds, see Trial Decision Criteria.

When a decision threshold is exceeded and criterion is met, the applicable oversight bodies (see Core Protocol) will consider the result and may determine a platform conclusion for public reporting. For example, an intervention may be declared effective or superior in a domain.

6.1 Platform conclusion procedure

The following high-level steps are involved in arriving at a platform conclusion:

1. interim analysis indicate a criterion has been met, see Trial Decision Criteria
2. results reported to DSMC for review
3. DSMC meet (as deemed necessary) and determine whether to report that a criterion has been met and recommend the implied action to the TSC
4. all participants enrolled up until the time when the action was enacted are followed up
5. a final (platform conclusion) analysis is run on the completed follow-up for those enrolled participants
6. the final analysis is used as the basis for further reporting

7 Trial Decision Criteria

Trial decision criteria are based on probability statements and are evaluated and used to direct the progression of the trial, see Trial Adaptations. Mathematically, the criteria are defined as the outputs of indicator functions applied to inequalities that define effectiveness, futility etc.

7.1 Effectiveness

At each analysis, the posterior probability that an intervention is effective (better than standard of care, see Treatment Contrasts) will be compared to a threshold of 0.99. If this threshold is exceeded then a trial decision of effectiveness will be made for the intervention and the standard of care treatment option may be dropped from the set of active interventions in the domain (allocation probability set to 0 for all regimens which include that domain standard of care, $d(1)$).

Table 1: Intervention effectiveness.

Decision	Comparison	Quantity	Threshold	Action
d_k is effective	d_k vs d_1	$\psi^d_{k1}(0)$	> 0.99	Drop d_1

7.2 Futility

At each analysis, the posterior probability that an intervention is futile (insufficiently better than standard of care, see Treatment Contrasts or insufficiently better than another reference treatment) with respect to a reference effect size of $\log(1.1)$ will be compared to a threshold of 0.95. If this threshold is exceeded then a trial decision of futility will be made for the intervention and the treatment option may be dropped from the set of active interventions in the domain (allocation probability set to 0 for the futile intervention).

The two contrasts of primary interest are the comparison of each treatment with the reference treatment $(\psi_{k1}(-\log(1.1))$, and where relevant, the comparison of combination of within domain treatments versus either given alone. For example, if treatment option l is the combination of treatment options k_1 and k_2 given together then the contrasts $\psi^d_{l,k_1}(-\log(1.1))$ and $\psi^d_{l,k_2}(-\log(1.1))$ may be of interest as additional futility checks for intervention l.

Table 2: Intervention futile.

Decision	Comparison	Quantity	Threshold	Action
d_k is futile	d_k vs d_1	$\psi^d_{k1}(-\ln(1.1))$	> 0.95	Drop d_k

7.3 Superiority

At each analysis, the posterior probability that an intervention is superior (in the best regimen, see Best Treatment (treatment in best regimen)) will be compared to a threshold of 0.99. If this threshold is exceeded then a statistical

decision of superiority will be made for the intervention and all other treatment options may be dropped from the set of active interventions in the domain (allocation probability set to 1 for the superior intervention).

Table 3: Intervention superior.

Decision	Comparison	Quantity	Threshold	Action
d_k is superior	d_k vs all d	φ_{dk}	> 0.99	Drop all d but d_k

7.4 Inferiority

At each analysis, the posterior probability that an intervention is superior (in the best regimen, see Best Treatment (treatment in best regimen)) will be compared to a threshold of $0.01/(K'_d - 1)$. If this threshold is not exceeded then a statistical decision of inferiority will be made for the intervention and the treatment option may be dropped from the set of active interventions in the domain (allocation probability set to 0 for the inferior intervention).

Table 4: Intervention inferior.

Decision	Comparison	Quantity	Threshold	Action
d_k is inferior	d_k vs all d	φ_{dk}	$< 0.01/(K'_d - 1)$	Drop d_k

8 Trial Adaptations

As the trial proceeds, some aspects of the trial status may change, for example randomisation probabilities are updated, new sites may start recruiting or treatment availability may change. Additionally, treatments and/or domains can be added or removed based on the trial results themselves, or due to information external to the trial.

8.1 Sequential Analyses

Analyses will be conducted frequently throughout the trial. The analyses will use all the data on participants who have reached the primary endpoint and have outcome data available to inform the current model. The results from the analyses, using the current primary model, inform updates to allocation ratios and statistical decisions.

The first analysis will be conducted after a minimum of $100 \times \max_d(K_d)$, that is, at least 100 participants per active treatment option within the largest domain have been enrolled *and* reached the 28-day primary endpoint. Subsequent analyses will be scheduled at fixed intervals (every 2 months) as long as the trial proceeds. However, if insufficient number participants have enrolled (< 50), the interim analysis can be postponed in increments of 1 month by notification to the DSMC. If recruitment is slower or faster than expected, there may be small or large changes in sample size from one analysis to the next, in which case the timing of analyses may be reviewed. The present schedule is adopted on the basis of logistics and pragmatically managing workload for the analysts, DSMC and personnel involved in data extraction and cleaning.

8.2 Actions Arising from Platform Conclusions

When a trial decision criteria is met, a platform conclusion may be declared and the following updates made. In some instances, despite a statistical decision being reached and a platform conclusion declared the following actions may be delayed. For example, if an intervention is found futile but further information is of interest for secondary outcomes, randomisation could continue.

8.2.1 Superiority

If a statistical decision of intervention superiority in a domain has occurred then, after review, a platform conclusion is declared and the intervention will be allocated with probability 1 at sites where it is available until a new intervention has been added to the domain. If the intervention is not available at a site then randomisation may continue to the non-superior interventions.

8.2.2 Inferiority

If a statistical decision of intervention inferiority in a domain has occurred then, after review, a platform conclusion is declared and the intervention will have its allocation probability fixed to 0 and will be dropped from the set of currently active interventions within the domain.

8.2.3 Effectiveness

If a statistical decision of intervention effectiveness in a domain has occurred then, after review, a platform conclusion is declared and the allocation probability to the domain standard of care option will be set to zero. If the effective intervention is not available at a site, then randomisation to the domain standard of care may still be allowed.

8.2.4 Futility

If a statistical decision of intervention futility in a domain has occurred then, after review, a platform conclusion is declared and this intervention will have its allocation probability fixed to 0 and will be dropped from the set of currently active interventions within the domain.

8.3 Response-Adaptive Randomisation

Following each analysis, the randomisation allocation probabilities will be updated to be proportional to the probability that each regimen results in the lowest log-odds of response among all active regimens, see Best Regimen.

The marginal allocation probability for each intervention will be determined and, in order to maintain a degree of power on the intervention comparisons with standard of care, the allocation probabilities will be corrected to achieve a minimum marginal allocation.

If active, the standard of care option within each domain will have a targeted marginal allocation of $1/K_d'$ where K_d' is the number of active interventions in the domain following the current analysis. If the domain includes only standard of care and one other active intervention, then a minimum allocation probability of 1/3 will be targeted to either intervention.

The allocation probabilities to regimens are updated as

$$\rho_j \propto \sqrt{\frac{\phi_j}{n_j + 1}}, \quad j = 1, 2, ..., K_A K_B K_C$$

where ϕ_j is the probability regimen j is best and n_j is the number having received regimen j.

The marginal allocation probabilities to each intervention, k in a domain d, are then calculated as

$$\varrho_{dk} = \sum_{\{j|d(j)=k\}} \rho_j$$

where $d(j)$ is the intervention from domain d that is a component of regimen j.

If the value of $\varrho_{d1} < 1/K_d'$, that is the allocation to standard of care in domain d less than target, or $\varrho_{dk} < 1/3$ (in the event where there is only one other active intervention) then the values of ρ_j will be iteratively adjusted until the target marginal allocation is achieved. The correction can be made according to the following rule, assuming that δ is the target marginal allocation

$$\rho_j = \begin{cases} \delta \frac{\rho_j}{\sum_{d(j)=k} \rho_j} & \text{if } d(j) = k \\ (1-\delta) \frac{\rho_j}{\sum_{d(j)\neq k} \rho_j} & \text{if } d(j) \neq k \end{cases}$$

If multiple domains need to be corrected then the above may be iterated over domains until convergence of the allocation probabilities. The corrected regimen allocation probabilities are then used to assign regimens to future

participants. The correction aims to maintain sufficient allocations to standard of care at the expense of potentially reducing the probability a participant receives the most probably best regimen at the time of their enrolment.

Once completed, these updates are communicated to the personal required to update the randomisation system, which will happen by default and not require formal recommendation by the DSMC. The updated allocation probabilities are communicated to the DSMC in the closed interim report.

Finally, we note that the randomisation system adopts the following policies:

- If a new participant is Ineligible or Unavailable for an intervention then any regimen involving that intervention will have $\rho_j = 0$ set for those regimens and the remaining values re-normalised to sum to one when randomising that participant.
- The randomisation process will use the same set of allocation probabilities for all sites, i.e. with no site level stratification. However, given the use of RAR, imbalance in number of persons per arm is expected.

8.4 Adding Interventions

When a new intervention is introduced into a domain, a run-in period will initiate fixed allocation probability of $1/K'_d$ where K'_d is the number of active interventions including the new one. This will last until at least 50 participants have been allocated to the new intervention across all regimens. Existing interventions in the domain will have their RAR allocation probability rescaled to sum to $1 - 1/K'_d$. Once the initial sample size has been exceeded the new intervention will be included in the RAR with all other active interventions.

New domains are introduced with balanced randomisation for each treatment within the new domain until at least 50 participants have been allocated to each new arm.

9 Reporting

Reporting covers considerations relating to internal (e.g. reporting results of sequential analyses to DSMC) and external reporting (e.g. reporting for publication in the academic press). The analyses identified in this document will be included in future trial reports and manuscripts. Exploratory analyses not necessarily identified here may be performed to augment the planned analyses. Any post-hoc or unplanned analyses not specified here will be clearly identified in any statistical reports and manuscripts for publication.

The DSMC has reviewed and accepted the interim reporting template.

9.1 Blinding

When publicly reporting the results of a statistical decision for a domain, the number allocated to each intervention and the number ineligible and/or for whom the domain was unavailable will be disclosed.

To maintain blinding to the performance of interventions in other domains, data on the proportions allocated to these other interventions will not be disclosed when reporting the baseline characteristics of participants in the reported domain.

9.2 Model Parameter Summaries

Where models have been used to inform inference, a summary of model parameters and the pre-specified posterior quantities of interest will be reported. At a minimum, for each model parameter we will report the mean, standard deviation, median, and 95% credible intervals (equal-tailed). Where a transformation is more appropriate, transformed parameters will be reported (e.g. odds-ratios, hazard-ratios etc.). Evidence of effects will be quantified by posterior probabilities over the relevant set of parameter values.

9.3 Participant Progression

When reporting on scheduled analyses (and other reporting when deemed necessary), a CONSORT-style flow diagram will illustrate patient progression through the platform and domains. Number (percentage) of participants randomised to each domain and intervention will be given for all randomised participants. Reasons for ineligibility will be presented by platform, domain, and intervention. Reasons for study withdrawal will be presented by intervention group.

Cumulative randomisation charts will be presented overall and by study site.

9.4 Participant Characteristics

When reporting interim analyses (and other repoting when deemed necessary), the following listings present data which will be collected at baseline. Descriptive summaries of these variables (counts and proportions for discrete, median and inter-quartile range for continuous) will be tabulated and presented in aggregate and by intervention.

Demographic:

- age (years)
- sex

- ethnicity
- weight (kg)
- vaccination status

Co-morbidities:

- chronic cardiac disease
- hypertension
- obesity
- chronic lung disease
- obstructive sleep apnoea
- asthma
- diabetes
- chronic kidney disease
- dialysis
- moderate or severe liver disease
- dementia
- malignant neoplasm in last two years
- iatrogenic immunosuppression
- smoking status

Prognostic factors:

- on room air for any of preceding 24 hours
- peripheral oxygen saturation (if on room air) (SpO2%)
- respiratory rate (breaths/minute)
- Glasgow coma scale (GCS) < 15
- highest recorded Urea last 24 hours (mmol/L)
- highest recorded c-reactive protein (CRP) last 24 hours (g/L)

9.5 Protocol Adherence

The protocol for intervention dosing are provided in the relevant domain-specific appendix to the core protocol. When reporting interim analyses (and other reporting when deemed necessary), adherence to the treatment protocol and protocol deviations will be summarised descriptively for each intervention.

9.6 Graphical and Descriptive Summaries

For each outcome, summaries will be presented in aggregate and by intervention group. Where longitudinal measures are available, cross-sectional summaries will be presented across time-points.

Dichotomous outcomes will be summarised by counts and proportions in each category. Ordinal outcomes will be presented using stacked bar plots, cumulative probability plots, and will be summarised by the frequency of each outcome category. Time-to-event outcomes will be presented using Kaplan-Meier plots and summarised by percentiles of the Kaplan-Meier estimates where available.

9.7 Missing Data

The number and percentage of missing data will be reported for all baseline covariates and outcomes. Where any values have been imputed, this will be reported along with the method of imputation.

References

Allison, P., 1982. Discrete time methods for the analysis of event histories. Sociological Methodology 13, 61–98.

Cox, D., 1982. Regression models and life-tables. Journal of the Royal Statistical Society 34, 187–220.

Joe, H., 2006. Generating random correlation matrices based on partial correlations. Journal of Multivariate Analysis 97, 2177–2189.

Lewandowski, D., Kurowicka, D., Joe, H., 2009. Generating random correlation matrices based on vines and extended onion method. Journal of Multivariate Analysis 100, 1989–2001.

Tokuda, T., Goodrich, B., Van Mechelen, I., Gelman, A., Tuerlinckx, F., 2011. Visualizing distributions of covariance matrices. Columbia Universiity, New York, NY, USA.
